# Gender violence among youth: an effective program of preventive socialization to address a public health problem

**DOI:** 10.3934/publichealth.2021005

**Published:** 2021-01-04

**Authors:** Sandra Racionero-Plaza, Itxaso Tellado, Antonio Aguilera, Mar Prados

**Affiliations:** 1 Department of Sociology, University of Barcelona, Spain; 2 Department of Pedagogy, University of Vic-Central University of Catalonia, Spain; 3 Department of Developmental and Educational Psychology, University of Seville, Spain

**Keywords:** gender violence, youth violence, sexual-affective relationships, effective interventions, peer group, preventive socialization of gender violence

## Abstract

Gender violence among youth is a worldwide public health problem. Youth is increasingly exposed to violence in sexual-affective relationships, both stable and sporadic, and the age of victimization decreases. This adverse life experience affects many areas of youth's life, such as education, social relationships and, especially, their physical and mental health, with consequences that can be very harmful in the short and long-term. This situation has given rise to many anti-violence programs for adolescents and youth, yet as some worldwide prestigious organizations, like the American Psychological Association, have pointed out, many of those programs do not work. In this article, we present a program of preventive socialization of gender violence addressed to adolescents that has proven effectiveness. The program was composed of seven interventions based on the social impact of the evidence on preventive socialization of gender violence. It was applied at a group level in groups of 15–16 years old teenagers in three high schools in Barcelona. The interventions were conducted over a period of one school year and shared the trait of discussing research evidence on preventive socialization of gender violence with the youth through egalitarian dialogue. These interventions have proved to have a preventive effect of gender violence victimization on the participating teenagers by raising their critical consciousness regarding a coercive dominant discourse in society that associates attractiveness and violence, supporting the transformation of their memories of violent sexual-affective relationships, and providing them tools to better analyze their and their friends' sexual-affective relationships along the lines of identifying gender violence and being more prepared to help others in this regard. The manuscript describes every intervention applied.

## 1. Introduction

Affective and sexual violence is a significant public health problem affecting millions of individuals around the world [1]. At the global level, the United Nations (UN) through its report “The World's Women 2020: Trends and Statistics” [2] shows that one in three women (35%) worldwide will experience physical and/or sexual violence by an intimate partner at some point in her life. Also, that younger women (aged 15–29) are at increased risk of experiencing intimate partner violence. Data from the latest report by the European Union in 2014, entitled “Gender-based violence against women: a European Union-wide survey” [3] showed that gender-based violence has a noteworthy prevalence, deeply affecting many women from the age of 15-year-old. For this report, 42,000 interviews were carried out with women from the 28 European member states, who were asked about their experiences of physical, sexual and psychological violence. Among the results, it stands out that: one in five women has been victim of physical and/or sexual violence by her current partner or previous partners; one in ten European women has been a victim of sexual violence (including both before the age of 15 and after the age of 15), and one woman in 20 has been raped. The prevalence in relation to psychological violence is even higher, with 43% of women who declare having suffered some form of psychological violence by their partner or a previous partner, having been humiliated in public up to 25%; one in 10 women has suffered harassment by their previous partner, and up to 23% had to change their email or phone because of such harassment. The data in the context of Spain are also very worrying. The latest national data comes from the “Macro-survey on violence against women 2019”, carried out by the Ministry of Equality [4]. 9,568 interviews were conducted with a representative sample of Spanish women, aged 16 years and over, who collected information on gender violence suffered at a physical, sexual, psychological control, psychological emotional and economic level. Putting our focus on the age range, the one corresponding to adolescents and young people aged 16 to 24, 19.3% of young women who have ever had a partner have suffered physical violence and/or sexual violence of any of their couples, 46.1% have suffered some type of psychological violence. One of the concerns presented from the survey data is that deeper analysis is needed to clarify the reasons why younger women show higher prevalence rates of gender violence. The consequences for the adolescent development of the experience of violent affective-sexual relationships are serious and multiple, affecting not only the victim but also her family, her friends from the educational center, her community and society in general [5]–[7].

Experiences of abuse and violence during adolescence can interfere with the normal developmental process, including the establishment of a stable self-concept and an integrated body image. There is robust evidence of the negative impact of violence in intimate affective-sexual relationships on the victimized girls' health [8]–[10]. Negative consequences from a psychological point of view include problems with body image, appearance of anxiety and depressive symptoms [11]–[13], suicidal ideation and/or attempts, poorer psychosocial functioning, and deterioration in self-esteem and psychological well-being [14]. Substance abuse, suicide risk [15] and eating disorders are also other mental conditions that can be suffered by adolescents. Longitudinal research has shown that gender violence victimization in adolescence is predictive of later depressive symptoms, especially dysthymic mood, hopelessness, sleep disorders, and anxiety [16]–[19].

While knowing the important negative health consequences of intimate partner violence at a young age is necessary, an intervention step is indispensable to address this problem. Indeed, so doing means responding to article 5 of the Convention on the Elimination of All Forms of Discrimination against Women (CEDAW), which pointed out that all states parties should take appropriate measures to tackle violence against women, including “to modify the social and cultural patterns of conduct of men and women, with a view to achieving the elimination of prejudices and customary and all other practices which are based on the idea of the inferiority or the superiority of either of the sexes or on stereotyped roles for men and women” [20].

Health literacy is one way to respond to such requirement established in CEDAW and, more broadly, to address the public health problem of violence against women. Health literacy has been widely studied in medicine and public health, and promoted to achieve positive changes on the population's health [21],[22]. It is defined as the ability to obtain, process, and understand basic information on health and necessary health services to make the most appropriate decisions [23]. Health literacy is of relevance for tackling gender violence among youth as a public health concern and is located in the field of interventions to prevent and respond to gender violence victimization among teenagers and youth. In this regard, there is international concern about the effectiveness of the many programs for prevention of youth violence that are being applied in high schools worldwide and the poor efficacy of most of them. Scientific organizations such as the American Phycological Association (APA) has made this concern public, stating that most educational programs applied in schools to prevent and response to violence are not working [24].

In Spain, many campaigns and programs do not work because they are not based on scientific evidence, they shift the focus of the problem and instead of discussing how boys and girls are socialized into attraction to violent males, they end up blaming the idea of having an ideal type of relationship in mind which is never violent, i.e., ideal love, as the cause for gender based violence among youth [25]. There is no scientific evidence that supports such statement, but the contrary is the case. Scientific studies have shown that romantic or ideal love protects from violence and benefits health [26]. By tearing down the possibility of an ideal affective and sexual relationship those programs only contribute to perpetuate violent affective and sexual relationships among youth as they promote sporadic sexual-affective relationships as an alternative when research has shown that there is much violence in such relationships [27].

Nonetheless, several intervention programs based on scientific evidence with social impact are showing excellent results in terms of gender violence prevention and response. In this article, we present a program of interventions on gender violence prevention and response addressed to adolescents and implemented in Spain which has already shown to be effective [28]. This program builds upon the evidence collected in many prior studies conducted within the research line on preventive socialization of gender violence [27], which is based on the dominant coercive discourse as one fundamental cause for violent sexual-affective relationships. The coercive dominant discourse [29] associate attraction to males with violent attitudes and behaviors, presenting them as more exciting. This discourse can be found in movies, television shows, songs, adolescent literature, social interactions, etc [30]. Adolescents learn the dominant coercive discourse in being exposed and involved in those media and artifacts, and those multi-faceted inputs put enormous pressure on them to engage in relationships that involve various kinds of violence.

The purpose of this article is to share the program of interventions applied with success with adolescents in Spain and which was grounded in the evidence with social impact from research on preventive socialization of gender violence. The program was implemented in the framework of research project entitled MEMO4LOVE [31] (2016–2020), funded by the Spanish Ministry of Economy & Competitiveness, which examined the change in mental models of attraction, memories and emotions via sustained actions of preventive socialization of gender violence.

## 2. Materials and method

### 2.1. The participants

The program was applied with a total of 131 adolescents (126 participated in all sessions), 71 were girls, 59 were boys, and 1 defined as fluid gender. The mean age was 15.4. The interventions took place in three high schools, during the academic year 2018–2019. The high schools were located in the urban area of Barcelona (Spain), mostly attended by students from families with low SES and highly culturally diverse. Two high schools were public and the third one was in part financially supported by the state. Interventions were implemented in natural groups of about 27 students, those were the already established class groups.

### 2.2. Recruitment

The participant adolescents belonged to the high schools that participated in the project. They were the students in the classrooms engaged in the project, which were all 4th courses of compulsory secondary education of the three high schools. It was the high schools the ones recruited for the project, and the selection was high schools in the metropolitan area of Barcelona (Spain) committed to implement an evidence-program of interventions to prevent gender violence victimization among youth over a period of seven months and comply with all the ethical requirements in the project. A number of schools were contacted and the final three were the first ones in giving a positive response. Parental informed consents and students informed assents were collected from all students in all classrooms before starting the project.

### 2.3. The intervention

Quantitative and qualitative data was gathered throughout the project in order to measure the impact of the intervention program upon the three dimensions related to gender violence studied in the research: peer interactions and dialogues, memories of sexual-affective relationships, and emotional responses/patterns of attraction. The qualitative data was collected via in-depth interviews and communicative focus groups with participant adolescents, and reports of memories of violent sexual-affective relationships. Quantitative data was collected using a questionnaire elaborated in the framework of the project focused on peer interactions and sexual-affective preferences; the Intimate Partner Violence Attitude Scale (IPVAS); and the Memory Experiences Questionnaire-Short Form [32] (MEQ-SF). All this data was collected in a pre-test before the beginning of the program for collecting baseline data and in two post-tests, one after intervention 3 and another after intervention 7. In addition, after each intervention a survey was used to gather the perception of the adolescents about the utility and impact of every session. The institutional review board of the Government of Andalusia (Spain) fully approved the research project, including its intervention part.

**Figure 1.**
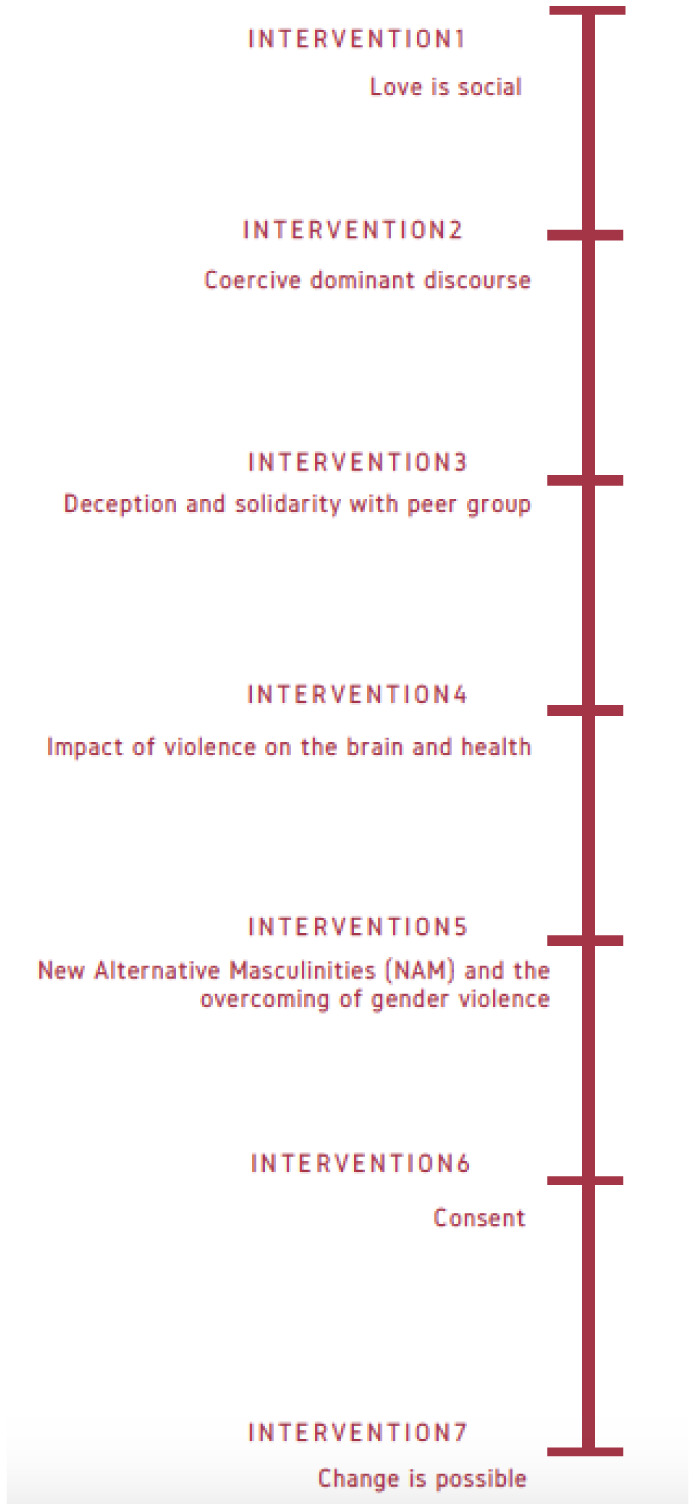
Plan of interventions.

The program was composed of seven evidence-based preventive interventions, every one focusing on a specific topic related with gender violence victimization from the perspective of preventive socialization of gender violence (see Figure 1).

The sequence was decided following the logics of the line of research of preventive socialization of gender violence, which starts from the notion of the nature of love and attraction, and the concept of dominant coercive discourse and its implications for gender violence victimization. The rest of interventions follow a logic toward transforming gender violence and fostering human agency to do so, while all interventions build upon each other.

The interventions were one-hour long sessions with a 15 to 20 minutes explanation which may include a power point presentation, a shared watching of a video or shared reading; all those activities aimed at providing the youth the scientific facts on the topics discussed. The sessions were delivered by members of the research group as well as by collaborators involved in gender violence prevention research. After the presentation, the session followed with a dialogue with the students on their believes, thoughts, agreements and disagreements, and impression of the content presented, relating it to their everyday life. Before the beginning of the first intervention, the adolescents were told that a crucial criterion for the participation in the sessions was never raising real names of peers present in the group or known by the group. Protection of all participants' intimacy was a condition for contributing to the dialogue and always ensured by the researchers leading the sessions. This was part of strong ethical principles that were at the core of the project.

## 3. Results

This manuscript shares the description of the program of interventions implemented in the MEMO4LOVE project. This article is not focused on the impact of the program, which is being already reported in other scientific articles [28]; thus the research data collected throughout the research project is not shared here. We share the program of interventions given the efficacy that actions grounded in the scientific evidence from the research line on preventive socialization of gender violence have already demonstrated [33],[34], including this program. Other publications [28]; have shown that this exact program of interventions contributed to raising the participant adolescents' consciousness regarding the presence of the dominant coercive discourse in their life, aided their better understanding of sexual-affective behaviors, thoughts and feelings, and supported the modification of sexual preferences in some female adolescents in a direction of rejecting violent males. The interventions also aided some participants' use of the knowledge gained in the project to help their friends and communities in reflecting upon coercive patterns of sexual attraction, the quality of their intimate relationships, and the different effects of toxic relationships on health. Some individuals reported leaving toxic relationships after the interventions. These results have been obtained following the research design, and the data collection and analyses procedures that were already evaluated by the scientific committee that approved the MEMO4LOVE project, as well as these findings regarding the impact of the interventions have been validated by the Advisory Council of the study and by members of the three educational centers participating in the project.

The 7 interventions of preventive socialization of gender violence were conducted on the following topics: 1) Love is social; 2) Dominant coercive discourse; 3) Deception in sexual-affective relationships and solidarity with peers; 4) Impact of violence on the brain and health; 5) New Alternative Masculinities (NAM) and its role in the overcoming of gender violence; 6) Sexual consent; 7) Change is possible and the importance of dismantling hoaxes. All these topics were always presented and discussed with scientific evidence.

### 3.1. Intervention 1: love is social

The first intervention revolved around the concept that “love is social”, that is, that mental and affective models of love and attraction are learned; they are not innate [30]. In order to give powerful tools to teenagers first was necessary for them to understand how individuals have been socialized over the years of their lives on feeling attraction towards specific types of persons, with movies, literature, TV series, magazines, peer talk, etc, playing a key role in such socialization process. The material employed in the session was first introducing the topic and then watching a short audiovisual document focused on the social nature of love and attraction [35]. Afterwards, guided group discussion took place for about 25 minutes. The questions posed for discussion after watching the video were: Is love and attraction an incontrollable ray of lightning or is it learnt? What do movies, series and music sell us that is most attractive? Can we do something to change the dominant discourse on attraction that is imposed on us? What can we do?

The video and the discussion made possible to deepen into experiences and knowledge on how love and attraction are not uncontrollable or invisible, but in fact these feelings are learnt.

The influence of media on boys and girl's socialization makes them believe that all what happens to them with regards to having love interests or feeling affective and sexual attraction is out of their control which has no scientific background, is completely discouraging and only contributes to misinformation of young people and adults. Learning about how individual's socialization around the globe tends to link attractiveness to people with violent attitudes and behaviors is key to unlearn dominant models of attraction. This new learning and the revision of past learnings must be conscious and intentional. Socialization in this coercive discourse (interpsychological level) leads to individual internalization (intrapsychological level) of cognitive and affective schemes where an association between attraction and violence resides, also reflected in corresponding neural circuits [36].

In the discussion, the young audience could express their learned ideas about the nature of love, bring into the table deterministic thoughts about it, and see with examples, often explained by the youth, that the type of person one's feels attracted to is the result of prior learnings. The discussion was guided to ensure treating the idea that attraction is learned and that given so, it means that when internalized models of attraction are toxic, for example leading to relationships with violent males, they can be changed [28]. A key take home message derived from the session was that no one is determined to have violent sexual-affective relationships and, even having had them, it can be transformed via the learning of new models of attraction free of violence.

### 3.2. Intervention 2: dominant coercive discourse

This intervention discusses freedom in sexual and affective relationships from the perspective of ending with being enslaved to the *dominant coercive discourse* [29] that links sexual attraction to men with violent attitudes and behavior. The material employed is a power point presentation created for this session and which gathers data from actual research publications on the dominant coercive discourse and gender violence among youth. Examples of songs, movies, literature, etc, showing the link between attraction and violence, all close to the reality of the adolescents are presented. This data illustrates the pressure that children, adolescents and youth experience from the media and sometimes from peer groups too to engage in intimate relationships, mostly sporadic, with people that despise them. The second part of the session is dedicated to guided discussion. Adolescents have the opportunity to see and reflect on the amount of physical and psychological abuse that teenagers suffer on their dates, as well as the type of abuses that young women can suffer in adolescence but also later in life [37].

This session makes clear that gender violence occurs not only in stable relationships, but sporadic relationships or hook ups are full of despise and different kinds of violence [27],[38]; this dismantles the hoax that gender violence only happens in stable relationships and that the alternative to escape from gender violence are hook ups. Indeed, the session clarifies that following the mandate of the dominant coercive discourse and submitting to it, engaging in sexual-affective relationships full of disdain is the opposite to freedom, but enslaving one's desire to the dominant coercive discourse [36]. An alternative model of affective and sexual relationships that overcomes double standards is presented, in which it is possible to have a relationship of ideal love with the same person in which there is passion and friendship, excitement and affection, craziness and tenderness [30].

Finally, the session ends with a dialogue on who are the individuals that they feel attraction for, why, which type of relationship do they dream and why, and how to achieve those dreams.

### 3.3. Intervention 3: deception and solidarity with peer group

In this session 3 topics are discussed and presented. First, solidarity with friends, second stop normalizing deception in relationships and third, overcoming double morality. In order to introduce these themes, short dialogues gathered from prior research data and excerpts of youth journals or media are presented. Mostly, they come from research on preventive socialization of gender violence reported in the book “Radical Love: A revolution for the 21st century” [30].

The first topic on solidarity with friends presents some dialogue among teenagers in which is presented how young people portray solidarity or insolidarity with friends who are not having a good time in their sexual and affective relationships. For instance, one friend finds out that other girl's boyfriend is cheating on her and she does not tell her friend. This just shows the lack of solidarity among women. Therefore, in a situation of betrayal, a friend may decide not to inform the victim because she feels that she is intruding since she is not part of that couple. Her actions of no intervention demonstrate a lack of solidarity with the victim.

In this case the questions for the group of students are: Do we show solidarity with the relationships of our friends? What do we do in a situation in which a friend of ours is not being well treated in the relationship? Finally, do we help, or do we keep ourselves out?

The second dialogue is regarding violence and the lack of respect in relationships. The question posed is: How do we act with friends that are experiencing such a situation? The questions then for the group after reading some excerpts of data portraying these situations in which someone is questioning themself to intervene or not because they may think that it's not their problem, the question for the participants are: With regards to the relationships that our friends have, do we think that some of the things that look like “normal” should not be happening? How could something like that affect the relationship? Finally, can we do something if this happens to a friend of us?

What adolescents learn after this session is that good friends intervene when a friend is being victim of violence, while always maintaining the freedom of the other person to choose whatever she or he wants. In general, this session deepens into the role of friends as upstanders.

The third dialogue discusses double standards [37]. Again, some excerpts of prior research data (quotations from adolescents) is presented to the students in which young people choose violent men for hook ups or short relationships thinking that the short temporality of that relationship might not be an issue if this is a violent relationship. While if they are seeking a long-term relationship, they will try to find someone who treats them well. This has been shown in the scientific literature [27].

For that reason, the participants are asked what do we seek in a relationship? Will that idea change with time? Do we seek people with certain characteristics for different type of relationships? One type for a stable relationship and a different type for a sporadic relationship? What is the consequence that might have in our friends if they follow double standards promoted by the dominant coercive discourse?

The fourth dialogue presented includes examples in which young women think that when being in a relationship with a boy who mistreats them, they will be able to change the behavior of that boy, they will be the one to “save him”. The questions posed to the participants are why sometimes some female adolescents think that they can change the violent boy who is mistreating them. Another question is why some girls still think they can change that boy even when the acts and behaviors of the violent boy do not change. Likewise, critical reflection is promoted regarding whether the girl's desire toward that aggressive boy would change if the boy changes his violent behaviors. The dialogues around all these questions can increase the adolescents' consciousness of the coercive dominant discourse in their life, more specifically, in their attraction patterns.

### 3.4. Intervention 4: impact of violence on the brain and health

The content of session number four is about the impact of toxic relationships on the brain and overall health. This session was one of the most well evaluated by the participant adolescents in terms of changing their prior ideas on (consequences of) gender violence and giving them tools to improve their relationships and better help their friends [28].

Data from research articles, including images of the brain, are presented, connecting toxic relationships with toxic stress which deteriorates mental and physical health, and the brain. In a stressed brain, cells change the brain architecture for the worse. Indicating that “comparable levels of adversity may lead to loss of neurons and neural connections in the hippocampus and in the middle prefrontal cortex (CFP)” [39]. In addition, this toxic stress also deteriorates physical health. Research shows that there is a direct effect of emotional states on immunity and illness. Quotes of women stating that they started to have digestive problems or being very weak as well as suffering headaches or suffering insomnia when being in a violent relationship, are presented. Research on the Telomere Effect is also shared [40]. Research has shown that stress-linked negative thinking patterns can damage telomeres—essential parts of the cell's DNA. One of the effects is premature aging. Biological manifestations of toxic stress can include alterations in immune function and considerable increases in inflammatory markers, which are known to be associated with worse health outcomes as diverse as cardiovascular disease, cancer, asthma, chronic lung disease, autoimmune diseases, etc [41].

On the other hand, the possibility side is also presented. Scientific evidence is shared showing that better relationships support better physical health, for example, having lower arterial pressure or better immune functioning as well as suffering less chronic pain. The take home message of this section of the session is the one from the Harvard Study of Adult Development, that quality relationships are the best predictor for a longer, healthier and happier life [42]. This data makes clear that romantic love or ideal love does not kill. On the contrary, it can save lives. What kills are violent affective sexual relationships. Also, friendship, as quality close relationship, is reinforced.

The group dialogue that follows the explanation of the research findings start with questions such as: Have you ever imagined these impacts of toxic relationships on health? What do you think now? What messages surprised you from the data shared today in the session?

### 3.5. Intervention 5: New Alternative Masculinities (NAM) and the overcoming of gender violence

In this session, different models of masculinity are presented. On the one hand, we have the dominant traditional masculinity (DTM) and the oppressed traditional masculinity (OTM), which are sides of the same coin and both promote gender violence, one (DTM) because it can be violent, and the other one (OTM) because positions itself behind DTM, as being of less value and feeling insecure in front of DTM and women. Data is shared showing that those two models of masculinity, and having such differentiation in mind, reproduces double standards. On the other hand, we have a New Alternative Masculinity (NAM) that overcomes gender violence [43], as it connects friendship and security as well as strength and bravery, and always takes a stand positioning himself in favor of the victim of gender violence and against the ones who are mistreating. In this new alternative masculinity, there is unity between goodness and attractiveness, and that is what helps to prevent gender violence. The presentation shows that NAMs do not fit in the double standard model, they do not belong to any side of that coin but break with that because of the union of goodness, truth and beauty in the same person. Romantic or ideal love, in any form, is only possible with NAMs and is presented as a revolutionary fact since having the dream of an ideal love is positive to fight gender violence.

The session concludes with a discussion guided by questions such as: Can we recognize examples of New Alternative Masculinities among the boys and men that we already know? Do we see these new alternative masculinities as much desirable and why? Which role does friendship have in not cheapening the dream of romantic/ideal love and to be able to walk towards the dreamed relationships?

### 3.6. Intervention 6: sexual consent

In session number six the main topic is sexual and affective relationships consent. A premise for an intimate relationship is that people take part in the relationship or sexual contact freely without any force. If there is no freely and willing choice, then there is coercion [44] and violence.

This session deals with this topic using a slide presentation. This presentation includes evidence-based information on “no means no” and, more importantly, what research has shown about the need of including more than verbal communication when addressing consent, such as nonverbal acts and contexts of power relationships [45]. For example, the cases of Anna Chambers in New York who suffered rape under police custody is presented, as well as the case of La Manada in Spain. With these examples, adolescents see that the concept of “no means no” is not enough. In fact, many people cannot say no because of the effects of any substance or for the paralysis produced by certain intimidatory situations, due to social hierarchy in our social structure or for any other circumstance. Likewise, it is also discussed with the adolescents that there may be circumstances in which asking and obtaining a “yes” does not prove that the desire one person seeks exists (for example, by the effect of the hierarchies underlying the relationship). For example, there is a power relationship between a student (18 years of age or older) and her college professor that might lead the student to say “yes” because of the coercion posed by the power relationship between them. Whoever has the greatest position of power in a relationship should always make sure that contact is desired by the other party [46]. All these situations are discussed with the adolescents using a text on consent and communicative acts published in *Diario Feminista* [47].

With these analyses and a guided discussion grounded in the aforementioned text, it is clear for the adolescents at the end of the session that it is necessary to introduce communicative acts, not only what is said but also the context and non-verbal language to ensure consent.

In addition, and importantly, the session clarifies that there is no free consent in a relationship between an adult and a person under the age of 18, and in some cases/countries, age 16. It is shared with the students that an adult who verbally or non-verbally proposes to a minor (under the age of 18) to have sex is coercing the adolescent. By definition, that is child sexual abuse. In such relationships, there is never equality or freedom, there can be no consent.

The following questions were discussed with the students in the second part of the session: What do you think about the information shared? Did you think saying “no” or saying “yes” was enough to secure consent? Do you know of situations where a girl or a boy has been “forced” to do something on an intimate level that they didn't really want?

### 3.7. Intervention 7: change is possible and the importance of dismantling hoaxes

In this session, two main hoaxes are presented. One is that “romantic love kills”, and the second one “the micro male chauvinism” (in Spanish “micromachismos”). Evidence is provided to show that none of them are not supported by scientific evidence and only serve to blame men and boys who are not violent and, in so doing, they foster gender violence among teenagers. The material employed in the session is a slide presentation and two texts [48],[49] also published in *Diario Feminista* and which deal exactly with these topics. This micro male chauvinist acts, instead of focusing on the violence being perpetrated on women, disorients the attention towards blaming non-violent men for small daily actions such as not putting the washing machine on properly, paying the bill, opening doors for women, etc. In this way, the attention on those small daily actions only serves to blame the non-aggressor men who position themselves against gender violence and who never commit any of the behaviors listed in the international definition of gender violence, behaviors that are performed by violent men.

A paper copy of the texts from *Diario Feminista* were distributed among the adolescents, read aloud, and then an open dialogue served to deepen on the ideas mentioned before.

## 4. Discussion and conclusion

Nowadays, UN's Sustainable Development Goals aim to achieve a better future addressing global challenges such as ensuring healthy lives and promoting the well-being for all of all ages (goal 3) and gender equality as a human right and necessary for a peaceful, prosperous, and sustainable world (goal 5). These goals pose one more framework to engage in the identification of effective programs to fight against gender violence among teenagers. This worldwide public health problem requires urgent transformative actions. In this article, we have shared a program of seven interventions framed by the research line of preventive socialization of gender violence that were applied as part of the MEMO4LOVE Project in Spain. We explain it here as the program has already proven to be successful in generating transformative changes in dimensions that are involved in gender violence victimization, as already reported and published in indexed scientific journals [28],[33],[34].

The program fulfills article 27 of the Universal Declaration of Human Rights (UDHR), which states that “Everyone has the right freely to participate in the cultural life of the community, to enjoy the arts and to share in scientific advancement and its benefits.” Every intervention of the program dealt with a key topic for gender violence victimization and response by providing adolescents with the scientific evidence about each of those topics, and then opening egalitarian dialogue to deepen into such evidence and relating it to their own experiences, thoughts and feelings. The positive impact of the program shows that adolescents can very well understand such evidence that they have the right to know it to improve their life and that of the people they love and care about. While this seems obvious, many programs of emotional education are far from being evidence-based and thus do not contribute to tackle gender violence among teenagers, as associations like APA have posed [24]. The program we report here is an effective response from the line of research of preventive socialization of gender violence, which had already demonstrated the effectiveness (social impact) of interventions grounded in the evidence collected in this research line [33],[50].

Also, the program was implemented in high schools, in the natural context of adolescents, indicating the important role that education and schools can play in contributing to respond and prevent gender violence victimization among teenagers but only if they apply programs grounded in scientific evidences with social impact. In making this real, schools will be meeting a right that all adolescents have around the world, that is, knowing scientific advancements about quality sexual-affective relationships and its health and social benefits. But not only. Serving the needs of adolescents and youth with scientific knowledge on preventive socialization of gender violence, schools and other educational and health institutions can open up possibilities for youth generations, if they freely decide to do so, to make choices in their life that raise their meaning and improve their health condition and that of their families, friends, and community. For the field of public health, the program reported here adds to existing knowledge on effective interventions to tackle violence in adolescents' sexual relationships, this being relevant as many programs of emotional education that deal with this problem do so without the ground of scientific evidence. In practice, the program shared becomes a tool for health professionals to tackle sexual violence in adolescents' relationships, and doing so in collaboration with educational institutions, thus fostering community to prevent and respond to gender violence.
